# The unmet need of personalized HCC screening—Lessons learned from the Swedish nationwide registries

**DOI:** 10.1002/ueg2.12239

**Published:** 2022-05-14

**Authors:** Simon Johannes Gairing, Jörn M. Schattenberg

**Affiliations:** ^1^ Department of Internal Medicine I University Medical Center of the Johannes Gutenberg University Mainz Mainz Germany

**Keywords:** HCC, liver cirrhosis, NASH, registry study, screening

Primary liver cancer is the third leading cause of cancer‐associated death worldwide and represents a global public health burden.^1^ Hepatocellular carcinoma (HCC) accounts for approximately 80% of primary liver cancer cases.^1^ While liver cirrhosis is the most important risk factor, the HCC risk is dependent on the underling etiology, gender and additional cofactors.^2^ All of these have to be consider when recommending surveillance strategies and assessing (cost‐)effectiveness.

In the current study, Bengtsson et al. explored the HCC risk in patients with liver cirrhosis in the Swedish nationwide health registry.^3^ The overall incidence rate of HCC was 23/1000 person‐years (95% CI 22–24) in 15,215 cases with liver cirrhosis. The cumulative incidence rate at 10 years was 12.2% (95% CI 11.6–13.0). Men had a higher overall incidence rate of 29/1000 person‐years (95% CI 27–31) compared to 14/1000 person‐years (95% CI 13–16) in women. The highest incidence rate 41/1000 person‐years (95% CI 38–45) was seen in patients with chronic viral hepatitis. In contrast, the lowest incidence rate (15/1000 person‐years (95% CI 13–16)) was observed in patients with alcoholic liver disease (ALD). Stratified by sex, men with viral‐related HCC had the highest (26.6%), women with ALD‐related HCC the lowest (4.3%) cumulative incidence at 10 years.

The strength of this study is the long follow‐up in the Swedish National Outpatient Registry that allows to assess the overall mortality and the impact of liver disease which is occurring slowly over time. The study provides valuable insights and is highly relevant for routine clinical practice. Current European guidelines recommend HCC surveillance for all patients with Child‐Pugh A and B cirrhosis independent of the underlying etiology, sex or age.^2^ In general, an incidence rate of ≥1.5%/year is considered to be cost‐effective.^2^, ^4^ Given the wide range of incidence rates that are further influenced by etiology, sex and age, the current study highlights that universal recommendations for HCC screening in cirrhotic patients will not have the same cost‐effectiveness across subgroups.

With the advent of personalized medicine at many levels, it is about time to implement this concept in HCC screening. Individualized surveillance concepts facilitating a risk‐based approaches can build on the data in the current analysis. An example for such an approach is the PAGE‐B (platelet, age, gender) score that predicts the HCC risk of patients with non‐cirrhotic chronic hepatitis B.^2^, ^5^ In agreement with previous studies, age was found to be an important risk factor particularly in patients with NAFLD‐related HCC with increasing rates in older patients: 3.26/1000 person‐years (95% CI 1.4–7.8) <50 years, 21.1/1000 person‐years (95% CI 16.7–26.5) in 50–65 years, and 28.9/1000 person‐years (24.1–34.5) > 65 years.^6^, ^7^ This could even be of greater relevance in NALFD patients, that are older compared to other etiologies.^6^

A total of 50.4% of patients had an ICD‐10 code of decompensation around the time of HCC diagnosis. This is a sad truth, as these patients will not be eligible for HCC treatment in most cases and the window of opportunity to diagnose HCC in an asymptomatic state has been missed.^2^, ^8^, ^9^ Based on the study design using ICD‐coding, hepatic encephalopathy and icterus could have escaped the authors when defining decompensated cases,^10^ and the lack of laboratory values – which allow to assess liver function ‐ is a limitation of this type of registry studies.

In line with previous studies, diabetes was found to be an independent HCC risk factor (HR 3.1, 95% CI 2.1–4.4). This is relevant given that diabetes is also an important risk factor of non‐alcoholic fatty liver disease (NAFLD) and the incidence of NAFLD‐associated HCC is expected to increase.^7^ In particular as NAFLD‐associated HCC can occur in the absence of cirrhosis^7^ and this requires that HCC surveillance strategies for this patients population need to be revisited. The lower incidence rates of HCC in NAFLD has a direct implication for cost‐effectiveness of surveillances programs.^7^ In additional barrier is the lower sensitivity of ultrasound in obese patients.^11^ In a recent analysis an HCC incidence rate of 0.8/1000 person‐years (95% CI 0.6–1.1) was reported for NAFLD (+/− liver cirrhosis) yielding a HR of 15.50 (95% CI 9.92–24.21).^12^ In the current study, the incidence rate was 21.6 (95% CI 18.8–24.8). In conclusion, the study by Bengtsson et al. provides important clinical insight and urges the field to implement personalized surveillance strategies for HCC.
